# "...because I am something special" or "I think I will be something like a guinea pig": information and assent of legal minors in clinical trials – assessment of understanding, appreciation and reasoning

**DOI:** 10.1186/1753-2000-3-2

**Published:** 2009-01-28

**Authors:** Michael Koelch, Hanneke Singer, Anja Prestel, Jessica Burkert, Ulrike Schulze, Jörg M Fegert

**Affiliations:** 1 Department of Child and Adolescent Psychiatry/Psychotherapy, University Hospital of Ulm, Steinhövelstr. 5, 89075 Ulm, Germany

## Abstract

**Background:**

The aim of this study is to assess and evaluate the capacities for understanding, appreciation and reasoning of legal minors with psychiatric disorders and their parents and their competence to consent or assent to participation in clinical trials. The beliefs, fears, motivation and influencing factors for decision-making of legal minors and parents were also examined.

**Methods:**

Using the MacArthur Competence Assessment Tool for Clinical Research (MacCAT-CR), an instrument developed for adults whose capacities to consent are unclear, we provided information about clinical trials and assessed understanding, appreciation and reasoning. We adapted this tool for legal minors and examined 19 children and adolescents between the ages of 7 and 15 with attention deficit/hyperactivity disorder (ADHD) or ADHD combined with oppositional defiant disorder (DSM-IV 314.00/314.01/312.8) enrolled in clinical trials. Parents were also examined using the MacCAT-CR.

**Results:**

Facts such as the procedures involved in trials or their duration were well understood by legal minors, but more abstract issues like the primary purpose of the trial were not understood by children and adolescents or by many parents. Legal minors also had difficulties understanding the nature of placebo and the probability of receiving placebo. Children's and adolescents' decisions were influenced by fears about their disorder worsening and by problems in their relationship with their parents. Parents wanted the best therapy for their children in order to minimize problems in school.

**Conclusion:**

Legal minors and parents need to be informed more precisely about specific issues like placebo and the primary purpose of trials. In general, the reasoning of children and adolescents was influenced by their experience with their disorder and decision making was based on reasonable arguments. Their fears were based on everyday experiences such as school performance or family relationships.

## Background

The involvement of children and adolescents in the decision-making process is an ethical prerequisite if they are asked to participate in clinical trials [1]. Although children and adolescents under 18 years in Germany (and in most countries) are legal minors and do not have the "legal capacity to consent" to research [2], children and adolescents have the right to information about research and to participate in the decision-making process, which is not necessarily connected to their competence to give legal consent [1,3,4]. The participation of legal minors is required and guaranteed by several guidelines, ethical codices and the law, even if choices patients make may be irrational, idiosyncratic, or unreasonable. It is a process of balancing the children's rights to decision making with their ability to deal with the responsibilities that come with it and with the responsibilities of parents for the decision making of their child [5]. Theoretical discussions about aspects of consent, assent and participation of legal minors and about the validity and conceptualisation of assent continue [6-8]. As psychopharmaceutical interventions have increased in recent years and clinical trials are more frequently conducted in the area of child and adolescent psychiatry [9], information for children and adolescents and their participation in the decision-making process will be a basic ethical need in the future. The question remains, how much do children and parents understand of the information provided about clinical trials in child and adolescent psychiatry and what is the best way to guarantee that the children's assent is meaningful?

Some studies have examined the capacities of parents in cases where their children participated in clinical trials e.g. in anaesthesia, oncology or surgery [10-15]. The results indicated that some parents had a high level of understanding of the study or treatment procedures, although a high proportion of parents showed no understanding of the risks and of the research nature of the clinical trials. Randomization, in particular, was not well understood.

Research on the participation of legal minors in the medical context is limited, but the few studies that do exist reveal that minors have well developed competences with regard to decision making in concrete treatment situations [16], that competences depend on the developmental status of the children and adolescents [17] and that confidentiality is important for young patients [7]. Most of these studies were conducted with somatically ill patients and few data are available in paediatric psychiatric patients. It could be assumed that psychiatric disorders influence subjects' capacities and competences in particular [18] and competences of children can change over time depending on developmental status. Furthermore, in some child and adolescent psychiatric disorders, conceptualization of the disorder by both parents and children may affect the reasoning of parents or children in decision making about treatment strategies or joining a clinical trial.

Abramovitch et al. [19] examined the capacity of children between the ages of 5 and 12 to consent to psychological research and concluded that, in general, children of these ages do have the capacity for meaningful assent to participation in research. Most of the children understood all or most of what they were asked to do in a psychological study, but few children below the age of 12 fully understood or believed that their performance would be confidential. Moreover, many children of all ages believed that there would be some negative consequences if they asked to stop. But this finding was inconsistent with the results obtained by Tait et al. [17] in children in surgical research. These children were not unhappy about annoying their doctors if they left the study.

Several authors observed a strong desire in children and adolescents to participate in the information and decision-making process on medical procedures, including standard medical care, even if the legal minors were unable to decide for themselves [20,21]. Children with psychiatric disorders or mental illnesses also wanted to be involved in the decision-making process [22-24]. There are two parts to participation: one refers to information (about the medical treatment/research project/clinical trial), which can increase the feeling of being involved [25-27]. The second aspect refers to the decision making itself (being involved in decision making/was the opinion of the children and adolescents respected?), which is reflected in whether or not the decision of the child or adolescent was accepted by parents and physicians [8,24]. But empirical data indicate that this is exactly where the deficiencies lie, namely in the information practices and involvement in the decision-making process, particularly of younger children [24,28].

As the competences of children and adolescents depend on the severity of their disorder, their developmental stage and other associated factors, these competences and factors have to be empirically examined. Which factors and arguments influence a child or adolescent suffering from paediatric and adolescent psychiatric disorders to assent or to refuse participation in a clinical trial? The most common disorders in child and adolescent psychiatry are attention deficit/hyperactivity disorder (ADHD) and oppositional defiant disorder (ODD). ADHD is characterized by impulsivity and inattention. The main characteristic of ODD is noncompliance with the instructions of adults. Both disorders may influence the decision making of children and adolescents and both disorders are commonly treated with psychopharmacological interventions. Therefore we wanted to examine whether it would be feasible to use an instrument developed for adults whose capacities and competences are unclear, and whether this would help to improve informing legal minors about studies in which they are asked to participate. In our study on children and adolescents with ADHD and/or ODD and their parents, we examined both the feasibility of providing information about clinical trials according to informed consent criteria and the understanding of information related to clinical trials. The appreciation of children and adolescents and their parents of what a clinical trial involved and their reasoning about participation in a clinical trial were examined. Furthermore, we analysed their attitude to health and their hopes and fears in relation to their disorders and their therapies. We wanted to shed light on the kind of arguments used by children and adolescents to give their assent or to refuse to participate in a clinical trial with medication. In addition, we examined the beliefs of parents about the necessity, advantages and risks of their child's participation in a clinical trial. To inform children and parents and to assess competences we used the MacArthur Competence Assessment Tool for Clinical Research (MacCAT-CR) [29,30].

## Methods

### Instrument

The MacArthur Competence Assessment Tool for Clinical Research (MacCAT-CR) is one of the standard international tools for the assessment and evaluation of competences for consent [29,31]. Both the MacCAT-CR and a similar instrument for treatment decisions, the MacArthur Competence Assessment Tool for Treatment (MacCAT-T), have been used in different studies in adults to evaluate capacities for decision making [32-38]. The MacCAT-CR is a semi-structured interview which relies on international criteria for informed consent (see also [30]). The MacCAT-CR is used to disclose relevant information to patients about their illness, treatment options in the study, and the risks and benefits of those options. The understanding, appreciation and reasoning of patients is evaluated in several parts and subparts. Participants are encouraged to explain the contents they have understood. The MacCAT-CR was adapted for children and adolescents using age-related terms and explanations. Interviews take about 35–60 minutes, depending on the need to repeat questions and the nature of the answers given by the participants. Interviews were conducted with the children and adolescents and their parents. The children and adolescents and their parents were interviewed separately. The feasibility of applying the MacCAT-CR in legal minors and scoring according to MacCAT-CR guidelines are reported elsewhere [30]. Whereas this instrument is mainly used to assess the capacity to consent in vulnerable populations, we used the MacCAT-CR as a tool to provide information and to assess and analyze the beliefs, motivation and fears of children and adolescents and their parents about participation in a clinical trial (see table 1). The complete interviews with the children and adolescents and parents were used for the analyses, independently of whether the answers given by the children, adolescents or parents were in line with informed consent criteria. The MacCAT-CR scores [30] were used to identify the answers which did not correspond to informed consent criteria as well as those that did. Both the corresponding and non-corresponding answers were analysed to illustrate the beliefs and motivations behind the formal criteria of informed consent.

**Table 1:** sections and subparts of MacCAT-CR

**Understanding**
Disclosure: nature of project

Disclosure: primary purpose is research

Disclosure: effect of research on individualized care (e.g. placebo, randomized design)

Disclosure: risk/discomfort (side-effects)

Disclosure: ability to withdraw



**Appreciation**

primary purpose is not individualized care
An explanation was e.g.:
"The doctors can explore whether the new medication works well in patients with an ADHD."

Reasonable possibility of less personal benefit (e.g. placebo)
Questions were e.g.:
"Are all the children in this study given the new medication?"
"Do you think you will receive a sugar-pill, a tablet without any agent?"

believes that the decision to withdraw will be honoured



**Reasoning**

consequential and comparing reasoning
"Well, what is your decision now?"

Generation consequences (e.g. on every day life)
In case of assent: "why have you made this decision, what are the reasons for your decision?" "OK. You say you want to take part. Why did you decide to take part? What were your reasons?"
logical consistency



**Expressing a choice**

Expressing a choice

### Participants

Two groups of children and adolescents were interviewed (19 children and adolescents in total). One group had attention deficit hyperactivity disorder (ADHD) alone and the other ADHD combined with oppositional defiant-disorder (ODD). Diagnoses were verified during examinations conducted at enrolment into the clinical medication trials (see below, K-SADS, SNAP were used among others) and diagnoses were made according to DSM-IV. For sample characteristics see table 2.

**Table 2:** Sample characteristics

**Study type**	**Diagnosis DSM-VI**	**Social status of the parents**	**N minors Sex**	**Age Mean (SD)**	**IQ Mean (SD)**
**Open-label trial with an extended-release methylphenidate preparation for ADHD**	5 *314.01, 2 * 314.00, 2 *concomitant 315.2, 1* concomitant 312.8	3 high 5 average 0 low	7 boys	13.3 (2.4)	104.1 (13.9)

**Placebo-controlled RCT with a new SNRI-preparation for ADHD + ODD**	12 * 314.01 + 312.8, 1*concomitant 315.2	2 high 9 average 1 low	12 boys	9.9 (1.7)	97.2 (14.7)

**Total** **Range**			**19**	**11.0 (2.3)** **7–15 y**	**99.8 (14.5)** **72–132**

The ADHD + ODD group was asked to join a randomized placebo-controlled trial in which an investigational drug (atomoxetine) was administered to treat the two disorders. Atomoxetine was not licensed in Germany at the time of the study. The children and adolescents with ADHD only were asked to join an open-label trial (licensed drug) using a long-acting methylphenidate preparation. 17 of the 19 children and adolescents had an average intelligence level (IQ 85–115, see table 2). None of the children or adolescents had experience with the study medication; nine children and adolescents were medicated with methylphenidate at the time of the interview and thirteen had prior experience with different types of psychotherapeutic medications.

19 parents were interviewed. Parents belonged to all social strata, which were classified using the standard clinical documentation (basic documentation of the German Society for Child and Adolescent Psychiatry and Psychotherapy) into low, average and high social status by employment (see table 2).

### Interviewers

The interviews were conducted by the clinical trial investigators (N = 3). One of the interviewers worked as a child and adolescent psychiatrist, and two of the interviewers were physicians in training for child and adolescent psychiatry. The interviewers were trained in conducting these interviews.

### Analysis

Each interview was audiotaped and transcribed. Interviews were independently analysed by two psychologists, who were not conducting the interviews. Both analysers were trained in assessing the interview.

Another analysis was done by rating the interviews but these results are reported elsewhere [30]. The attitudes of children and adolescents and parents towards health and their concept of clinical trials were investigated. The answers were analysed using qualitative methods to gain insight into the decision-making process of children and adolescents. Analysis was conducted in a similar way to qualitative content analysis [39,40], but some modification was necessary as categories of contents were already provided by the MacCAT-CR (see table 1). First we searched for parts which were not well understood by the participants. Then answers were searched for the sequences, in which children and adolescents and parents explained their decision making and the rationale for their decision. Analysis was not restricted to explanations that were in line with informed consent criteria.

## Results

In our study, the parents in particular showed a strong desire to speak during the informational conversation about their child's problems, the patient's history and former therapeutic interventions which were effective or which failed. Furthermore, parents were interested in speaking about the present impact of the disorder on their child's ability to function in every daily life.

Children and adolescents were asked whether they were content with the interview. They were interested in talking about the study, but all of the 19 participants were bored and annoyed by being repeatedly asked the same questions, as is required by the MacCAT-CR for evaluating capacities.

After the interview, 10 children and adolescents from the group with combined ADHD and ODD assented to their participation in the study. In the group with ADHD only, 5 minors gave assent, one minor refused and one was undecided. 18 of the 19 parents interviewed consented.

### Understanding

Altogether, 10 of 19 of the children and adolescents were able to understand the information about the trial very well, 7 of 19 understood the information at a large extent but showed some deficiencies in understanding and only 2 of 19 children had a completely insufficient understanding. Issues which were very well understood or which were not well understood by the participants are described following more detailed. Except the named two participants, the children and adolescents showed sound understanding of study procedures such as study visits, duration of the trial, blood drawing, being sober and taking medication etc. The same 17 of the 19 children also had a very precise understanding of possible side-effects. But most of the children (n = 13) failed to understand that the primary objective of the clinical trial was research and not the best individualized care and personal benefits. Even parents did not perform well in understanding this fact.

In addition, in the placebo-controlled clinical trial, the nature of placebo and randomization seemed to be too complex for the children and adolescents to understand. This part was not well understood by two thirds of the children and adolescents and even a small minority of the parents. Whereas children and adolescents understood the term "sugar-pill", the significance and nature of the placebo was not understood. Furthermore, children and adolescents underestimated the possibility of receiving placebo. Most, but not all (16 of 18), parents understood the nature of placebo although half of the parents did not comprehend the significance of randomization.

Both parents and children and adolescents clearly understood that they were not forced by physicians to participate and that they could terminate participation at any time. The two children with a sub-average IQ performed more poorly than the other children in understanding, and also in all further sections of the MacCAT-CR (see also [30]).

### Appreciation

Especially in the part of the MacCAT-CR where "appreciation" was evaluated, children and adolescents performed inappropriately to criteria as required by the MacCAT-CR.

A basic element of informed consent or assent is a subject's appreciation that he or she is asked to participate in a study for the purposes of research, and not to receive the best individual care. MacCAT-CR tests this appreciation by asking the participants why they think that they have been asked to participate in the clinical trial. Children understood that the efficacy of a medication has to be tested, but there were misconceptions about why efficacy has to be tested, which were not directly correlated with the age of the children. 16 of the 19 children thought that the trial would examine whether the medication would help them:

"So that I feel better and just generally."

Boy P, 12 years old

"That it helps me (...) and whether it helps other kids."

Boy R, 10 years old

"Because if it (the medication) is not effective, and the medication is sold and the doctor in the pharmacy...ehm, if the medication does not work then, then people buy it and then they spend their money for nothing."

Boy S, 9 years old

For almost all children as well as for more than one third of the parents, their appreciation of the primary objective of the trial (which was research) was that they would derive personal benefit by joining the trial. The fact that the primary objective of the trial was research was not really appreciated by the children. The children and adolescents in particular did not consider that the aim of the question was to make them think about the altruistic aspect of research participation. It was not the participants but the researchers who would primarily benefit by conducting the trial, whereas the benefit to the patient was questionable. Children thought they were asked to participate in the trial because they would personally benefit from this. They explained that, from their personal point of view, they expected benefits for themselves. For example, a handful of the children and adolescents said that the personal benefit they expected from participation would be that they were one of the first to receive a new treatment or medication. They appreciated the new medication and believed it to be the best and most innovative therapy.

One boy had a realistic attitude about the trial. He said that many children were necessary because the effectiveness of the medication was not proven. One boy answered the question about what role he would play in the study correctly:

"*I think I will be something like a guinea pig*."

Boy H, 12 years old

Therefore, he appreciated correctly that he would join a trial where the primary purpose was research but he had a misconception about why he was asked to join. The same boy's answer to the question of what the primary objective of the trial could be and why he was asked to participate in the trial was:

"*Because, I'm something like a special case, because I'm really intelligent, I'm suffering from ADHD and at the same time from ODD and I also have dyslexia and because of me being a mixture of nearly everything (...)*."

Boy H, 12 years old

Another boy, very young, offered altruistic reasons for why he was asked to join the trial:

"And, that it will improve the disease in others, and that it (the medication) also helps other children, because it should also help other children, not only me."

Boy S, 8 years old

A mother expressed her appreciation of the primary objective of the trial in very realistic terms:

"We can benefit from it, you can benefit from it or we can all or both go down the drain with it."

Mother of boy K (10 years old)

While appreciating the nature of placebo and the possibility of receiving placebo, participants shared with us their personal beliefs about why they or their children received placebo. They applied placebo to their own personal situation. For instance, one of the participants thought placebo was dispensed to prove whether he really suffered from ADHD and was not pretending. No child appreciated that there was a fifty/fifty chance of receiving placebo.

The perception of whether they would receive placebo or not was driven by the children's beliefs, and not by a realistic balancing of the chances.

Interviewer: "Who decides whether you will receive a placebo?"

„The psychologist"

Interviewer: (...) "Do you think you will receive a placebo?"

"Not really"

Interviewer: "Why not"

"Ehm because, in fact, I don't know, I don't have a reason."

Boy S, 8 years old

In response to the question whether they thought they might receive placebo, all the children and adolescents believed they should first explain why a placebo is used in a study. Their appreciation was that another child could receive placebo, but not they themselves. One boy said that he thought there was a placebo control in the trial and that nobody would know whether he received a placebo or not.

"ehm I will probably receive a sugar pill between visit two and visit five, you won't know that, my mother won't know, and I won't know either, and nobody else will know. And what might happen is that I will be a little bit fidgety, that I will be fidgety and, ehm, on visit five I will receive the true medication (...) (I will receive the placebo) to explore, ehm, whether the new medication, if this medication is superior, as if I take no medication, or if I take the new medication."

Boy L, 10 years old

According to him, the purpose of placebo was to help clarify whether the new drug would be superior to taking no medication. This would appear to be correct, but he went on to say that if he did receive placebo he could develop some symptoms of ADHD and therefore he and the study physicians could clarify whether he needed a medication or not. He believed that the placebo would be given to him to clarify whether it would be better for him to take any medication or no medication.

Parents also had some difficulties with appreciating placebo. A mother thought that placebo would be given to test whether the medication really worked for her son. Another mother thought that placebo would help clarify whether her son really suffered from the disorder or whether he needed more attention from his family. This mother's answer to the question of whether she believed that her child could get a placebo was as follows:

"*Perhaps, I imagine that what will be tested is if he needs it (the medication) or if he really just (needs) attention!"*

Mother of boy H (12 years old)

The MacCAT-CR stresses possible misconceptions by patients who think they will not receive any treatment if they refuse to participate in the study, or that the physician will be angry or that this decision would harm the physician-patient relationship. According to MacCAT-CR, competent appreciation means understanding that the consequence of refusing participation will lead to the patient's receiving another treatment. One boy's answer about the consequences of refusing participation, which was typical for half of the children, was:

"If I do not want to join from the beginning, then Dr. XY (former therapist) continues to care for me, and then, if I want to participate, you will treat me, but I do not have to participate."

Boy K, 10 years old

In our study, the children and adolescents were troubled or thought that their disorder was becoming worse and as a consequence that they would perform worse in school or would have problems with their parents if their oppositional defiant disorder symptoms became worse. Many of the children and adolescents had prior experience with medication or psychotherapy and the effects of this medication/psychotherapy were often unsatisfactory or disappointing. Therefore, it is understandable that they did not refer to the alternatives represented by medication or psychotherapy. However, it is impossible to rule out that some children and adolescents thought that the only available medication would be the study medication.

Whereas it was very difficult for children and adolescents to deliberate on the primary objective of the trial, they were able to appreciate fully the benefits, risks and consequences for every day life. Neither children and adolescents nor parents thought that a potential decision on their part to refuse to take part in the trial would have negative consequences on the relationship between themselves and their physicians or on any further treatment in the department.

### Reasoning

All children and adolescents indicated that the reasons why they decided to participate were that they wanted help with the problems related to their disorder: problems in school, problems with friends and with their families. Children and adolescents argued that if they did not participate, they would worry about performing worse in school and would experience problems with their parents and families because of behavioural problems. One boy indicated that he would participate because otherwise he feared he could become more aggressive and hurt his mother. His reason for deciding to participate was that he wanted to improve and change his disruptive behaviour. He thought his behaviour was not fully under the control of his own will, which was a view also expressed by about half of the 19 children:

"Then it will get worse (with my symptoms), or well I think that it will get worse, because it got worse every year, ehm, I will grow up, will be stronger, and then puberty will come as well, so I think, I should participate, well for my mother's sake and even for my sake."

Boy H, 12 years old

Other boys made the following statements:

"Because I believe, I believe that with the new medication everything will be much better (...) I just have faith in the new medication."

Boy L, 10 years old

"I will just take it, I'll go along. I will just try it and see how it goes. (...)Well, so I can see how it will work and so on and if I will be able to concentrate a bit then."

Boy K, 10 years old

"Yes, because I am interested to see if it helps me."

Boy B, 10 years old

Children with ADHD combined with an ODD did not differ from those without an ODD in their hopes that study participation, or the study medication, would help them to reduce problems in their families and with peers. In this sample, the ODD did not lead to more conflictual relationships with parents on the issue of study participation and therefore children with an ODD were no less willing to participate.

Both parents and children indicated their high expectations of the effectiveness of the new medication and their decision to join the trial were driven by fears of anticipated problems in school and at home if they did not try a new (and supposedly better) therapy. The failure of prior therapeutic interventions was the main reason for parents to decide that the child should participate in the trial. A mother made this statement:

"Well, we're not happy with the effects of previous therapies, there are problems in school, and we want to help him with the best option. Therefore we see this (participation in the trial) as a way of doing this."

Mother of boy U (8 years old)

"I hope that the study will bring some positive effects for (my son) and we can optimize the therapy."

Mother of boy F (11 years old)

As one perceived advantage of the study medication was the fact that methylphenidate was supplied in an extended-release formulation, six of seven parents and three of seven children and adolescents incorporated this expected advantage into their reasoning. The hope that the medication would be more effective because of the element of constant release was indicated as one reason influencing their decision. Furthermore, the fact that the new medication was easier to handle, in that it had to be taken only once a day and not twice or three times daily as was the case with the licensed preparations for ADHD, was seen as an advantage and a reason for participation. Six of seven parents, as well as 3 of the 7 children and adolescents asked to use a long-acting methylphenidate preparation, recognized that the once-a-day dosing decreased the risk of forgetting to take the medication and was a very important advantage as it made it possible for the children and adolescents not to have to take the medication in public (e.g. in school). Therefore, children and parents thought that once-a-day dosing removed the stigma associated with having to take the licensed preparation in public.

"One reason for me is definitely that I can forget it (the regular, not extended-release methylphenidate) and on the other hand I am a little bit interested in it and how it will be then (the extended-release methylphenidate)."

Boy G, 15 years old

Further advantages were seen in the hope (or therapeutic misconception) that participation in a research trial would supply the children and adolescents with the best and most innovative medication, which would not be available elsewhere. In 10 of the 19 cases, the decision was driven by the failed efficacy of previous medications.

Side effects were not always anticipated to be very negative by children and adolescents. Two boys stated that there were benefits from suffering mild side effects such as bellyaches or nausea: they would not have to go to school. One very young boy estimated that the side effects would not bother him very much:

"I may get stomach troubles, but never mind."

Boy T, 8 years old

Blood drawings and examinations were expected to be disliked and one boy argued that he was afraid to have his blood drawn and therefore did not assent to participate.

"I don't want to join. (Why?) Due to the blood drawing, (...), yes, this would be the worst for me. "

Boy U, 8 years old

When children or adolescents participated in trials, a different physician from the research unit cared for them and they no longer saw the one who had previously given them routine care. Therefore, one child was afraid of losing his therapist with whom he had a good therapeutic relationship. More than half of the children and adolescents felt bothered by the need to make frequent visits to the study physician for the purposes of study examinations. The large amount of time needed to participate in the study was weighed up by the children and adolescents. The children who did not assent found the balance to be negative:

"I do not want to participate, because I want to continue with my group (therapy), there I know many of the other kids, and here I know nobody, (...) there all my friends go to the group. There (in the group therapy) my other friends also attend, from my school, my friends from my previous school attend. And even if the group ends now, we can still go there in the future. (...) Also, my old medication did work a little bit and improved me. My leisure activities would be worse, if I had a visit on Friday or Tuesday, that's when I always have handball training, all day, and Thursday, that's when my grandmother comes to us, therefore I cannot come to the department, and on Wednesdays, then my aunt comes to see me. My aunt and sometimes my cousin or it is a family evening or something like that. (...) I can't quit handball and I can't miss seeing my grandma, I can't."

Boy S, 8 years old

The children and adolescent who assented considered the time that participation would involve but were able to balance it out with the expected benefits.

"Well, I do have a lot to do but I would fit it in."

Boy E, 13 years old

10 of the 19 parents stated that one disadvantage of the clinical trial was the large amount of time involved. For example, they mentioned difficulties such as long car journeys to the clinic, less leisure time or less time for sports activities for the child. When parents refused to allow their child to participate in the study, the reasons for refusing included that it was too great a commitment (above all in terms of time) and fear of giving an investigational drug to the child over a long period of time. Although they gave their consent to participation in the study, other parents and children also stated that they had concerns about participation. Overall, the main fear about joining clinical trials was the fact that their child would receive an investigational drug over a long period of time.

One mother indicated fears that the new medication might deliver a higher dosage than needed – which was indeed the case. As the study medication was administered at fixed doses, a higher dosage per kilogram body weight was prescribed.

Two third of the children and almost all parents did not think that refusal to participate could bring negative consequences on the level of health care or personal dependence on the physicians. They reasoned that personal negative consequences would be in terms of poor school achievements, arguments with their parents etc. But none of the children or parents expected negative consequences in the health care setting or in their personal relationship to their doctors. This may be related to the German health care system, where access to care and availability of medication, psychotherapy and child and adolescent care is almost entirely guaranteed.

This study identified some categories of arguments and considerations on the part of the children that induced them to participate in a study or to refuse participation (Table 3). Hope that their symptoms and, in somewhat more abstract terms, their behaviour would improve were aspects which motivated children and adolescents to assent. Both the personal benefits of improved symptoms (e.g. better concentration in school, fewer arguments with peers) as well as the benefits for their parents and families were considered by children. Furthermore, the expectation of a more user-friendly medication taken no more than once a day was a reason for children and adolescents to assent and to undergo the examinations of a clinical trial. The chance to test something new represented a third group of reasons which motivated children to assent. Most of the children wanted to test the new medication to see whether it would work for them. This could be regarded as a sort of sensation-seeking behaviour or a type of explorative behaviour, but it also indicated their experience of treatment failures in the personal history of their disorder. They balanced the option of being the first to test a new and maybe better medication against the risks and decided for participation in the trial, although the children also appreciated the disadvantages and the risks of the trial.

**Table 3:** Motivating and discouraging factors for children to participate in clinical trials

**Motivating factors**
- Hopes for improvement of their own behaviour based on experience
With benefit for themselves
With benefit for their families
- Comfort (once a day vs. several times a day)
- Explorative behaviour/sensation seeking

**Discouraging factors**

- Changes in treatment settings
- Time spent
- Study examinations, especially invasive exams/blood-drawing

Factors which influenced children to refuse assent to participation included changes in their treatment settings with new physicians and loss of their familiar therapists and peers from their group therapy. Furthermore, the high amount of time spent on frequent visits to the hospital was a reason which discouraged assent. The third category of reasons for not assenting were the study examinations, especially invasive medical procedures such as blood drawing which can be expected to scare children.

### Performance of children and adolescents with ADHD and with ADHD combined with ODD

We found that four children from the group with ADHD and ODD had a worse understanding compared with other children. Therefore, understanding was poorer in the group of children and adolescents with the ADHD combined with ODD and their answers were less differentiated compared with the group of patients with ADHD alone.

Three of these children had either a lower IQ compared with the total sample or no experience with medication before entering the study or both. One of the children had an average IQ and experience with medication. As the sample size is small and all factors are applicable, none of these factors can be validated as the essential reason for the poorer performance in the MacCAT-CR. But it may be seen as a trend that IQ and experience influence understanding and reasoning, whereas a conflicting relationship that is due to externalizing disorders seems to have no negative influence on willingness to participate.

## Discussion

This study revealed that legal minors demonstrated a large number of competences. Procedures and issues related to study examinations were very well understood by children and they also performed very well in appreciating advantages, disadvantages or discomforts caused by study procedures. But more abstract subjects like the primary objective of the trial were not well understood and appreciation was low – by both children and adolescents and often by parents. The capacity to understand the nature of placebo was low and children and adolescents had an inadequate appreciation of the probability of receiving placebo. The study revealed some indications of therapeutic misconceptions in children as well as in parents when joining the trials.

These results correspond to prior findings which reported that children and adolescents understand concrete treatment procedures very well [41], but that children below the age of 12 or 9 years had problems with understanding and cannot be expected to consent or assent to clinical research in a meaningful way [19,42]. If children younger than 12 years have fears about negative consequences if they ask to stop participating in studies as was reported [19], then a prerequisite for a meaningful assent or consent is not fulfilled. In our example, children and adolescents really felt free to withdraw from the study and indicated that they had no worries about any negative consequences with regard to doctors, which is consistent with the results obtained by Tait et al. [10,11]. Therefore, children with a psychiatric disorder also seem to understand one essential element of informed consent, the autonomy of the decision to join or to quit a trial. Furthermore, they balanced the potential disadvantages, for instance the expenditure of time on study examinations against possible advantages, such as once-a-day dosing or more effective treatment, which indicates meaningful reasoning. According to the responses given by our participants, it is reasonable to conclude that some issues required by informed consent can also be fulfilled by legal minors with psychiatric disorders.

But we found that some fears and worries which impacted on the decision-making of children and adolescents were not immediately related to study withdrawal with regard to the informed consent paradigm. The children and adolescents in our group worried about their disorder worsening, about increased problems in school and poor school performance, or they were afraid that their behaviour would get worse if they stopped the study medication. It appeared that the search for a satisfactory treatment option made children and adolescents prepared to accept certain discomforts such as the study procedures. As paediatric psychiatric disorders impair social functioning and also impact on children's families and social circle, these more abstract and inter-personal aspects that the children expressed as motivators for study participation seem to be particular to child and adolescent psychiatric patients, who join clinical trials to improve their complex impairment.

The possible adoption of parental wishes was not directly expressed by children. If children really assumed that their parents wanted them to join the trial, they did not directly communicate this in the interview as being an influential factor in making their decision. But children and adolescents stated that parents feared that their behaviour could worsen if they did not join the trial. As a consequence, the implication is that children and adolescents feared that deterioration in their behaviour could cause problems in their interaction and relationship with their parents. This may reflect a special kind of "altruistic" motivation in children for participating in research projects on child and adolescent psychiatry. But it is reasonable to assume that the motivation to try a new medication would not change if the same medication were offered in routine care. Therefore this "altruistic" motivation on the part of children would not differ from the general motivation of children with behavioural problems to undergo any treatment to improve their behaviour. We did not observe that the presence of ODD was directly connected with a more negative attitude in the children towards clinical trials. On the contrary, fears about the negative consequences of their disorder even made them prepared to balance discomfort against the possible benefits of the study medication.

The difficulties we observed in the study that children and adolescents experience in appreciating complex research concepts may imply that they need protection if they are involved in research projects. Parental consent is needed to protect children, as they remain vulnerable, even though their factual understanding is good. Otherwise, the partial competences shown by the legal minors, whom we observed in the study, should encourage the development of shared decision making in these issues, which would guarantee the participation of children and adolescents in decision making while respecting their free will [5].

Additionally some parents lacked competences. There have been previous reports about therapeutic misconceptions held by parents or patients in general if they or their children were asked to join a trial [9,43]. In these studies, less extensive information methods were used compared to our study, but even the application of a very complex and detailed method like the MacCAT-CR cannot avoid therapeutic misconceptions by parents or children. We detected these misconceptions in our study as well: in particular, the children, in general, did not appreciate that they would not receive the best proven treatment. Nevertheless, the children reasoned that they were asked to participate to test whether this medication worked, so they understood that there was no guarantee that the study drugs were effective. The practice of providing information to parents, children and adolescents in clinical trials or studies can be improved by using structured interviews. This increases understanding, even that of younger children. The discrepancy between clinical (all parents and children were judged to be competent to join the studies) and formal evaluations (in which doubts are raised whether all subjects are competent) points to the influence that the procedure used exerts on competence judgements, and also demonstrates that a more formal evaluation can help to identify patients who need further information. As the aim cannot be to obtain informed consent in a legal meaning from legal minors or young children, the primary target of clinical investigators in clinical studies with an ethical position should be to improve information for minors and not to obtain a "formal" informed consent. Parents should be informed in precise terms that the primary purpose is research, and also why this is the primary purpose. Researchers should be aware of the fact that not all parents understand information sufficiently well, and information should stress the importance of identifying these parents and patients so as to provide iterative and clear explanations and information for them. Using a suitable instrument can help to detect misconceptions. We would not suggest using the present form of the MacCAT-CR routinely to obtain consent or assent, but it is a clear strength of the MacCAT-CR that it reinforces children's and adolescents' reasoning about their decisions and it provides reassurance about their comprehension of their decision to take part in a study, which is an essential part of each "Informed Consent", as well as being essential to the investigator's assessment of the subject's comprehension [30]. Therefore, this instrument should be further developed and modified for the use in children and adolescents.

In conclusion, the motivating factors reported by children in this study demonstrate that further studies are needed to examine the attitudes of children towards clinical research and to analyse in more detail and in different studies what motivates children to take part in clinical trials and whether the severity of their disorder influences their attitudes and willingness to participate in research projects.

### Limitations

This was a feasibility study and therefore the sample size was small. Results may be different in studies with children and adolescents who have other paediatric and adolescent psychiatric disorders, such as anxiety disorder or schizophrenia. The clinical trials differed in their study procedures: one was a randomized trial, but not the other. One trial included invasive study procedures such as drawing blood, fasting etc., the other did not.

## Conclusion

Even if children and adolescents showed some deficiencies in understanding and appreciating the nature of a clinical trial and its primary objective, these results must be related to studies with adults, which also found that up to 60% of adults demonstrated problems in understanding the contents of the information about clinical trials or seemed unaware that treatment was randomly determined and different from personalized care [44]. The children, adolescents and parents in our study also showed poor performance in appreciating the primary objective and in understanding the meaning of placebo. Clinical investigators must be aware of these deficits and misconceptions and improve information about these issues. Hopes of an improvement in behavioural symptoms and of better social functioning motivated both the legal minors and their parents to assent or consent to study participation. IQ and age of the children influenced understanding, but basic elements of the "informed consent" paradigm, such as making the choice freely without negative consequences for further treatment, were understood by almost all children. The therapeutic misconceptions we observed were related to receiving best individualized care or the hope of being selected to test whether the study medication worked well in the single, individual case. Although, in our study, children, adolescents and parents received information leaflets about the clinical trial prior to verbal discussions, the problems of understanding described above still existed. Even if the MacCAT-CR is an instrument which may improve information about clinical trials in children by structuring the information process, additional or alternative methods of information about the nature and probable consequences of clinical trials are needed. The MacCAT-CR helps to identify therapeutic misconceptions and can also assist in providing more precise information to these patients with misconceptions. The results emphasize both the need to protect children involved in clinical trials by the requirement for parental consent, and the need for shared decision making about participation in clinical trials by children and parents.

## Competing interests

This study was supported by a grant from the Eli Lilly International Foundation to MK. There are no other conflicts of interest. MK received further research grants from the German Federal Ministry of Family Affairs, Senior Citizens, Women and Youth, the Federal Justice Department of Switzerland, Boehringer Ingelheim, European Academy. He has acted as clinical investigator in trials performed by Astra Zeneca, Janssen-Cilag and Eli Lilly. Travel grants were provided by Janssen-Cilag, University of Rostock, DGKJPP, UCB and some welfare institutions.

JMF received unrestricted research grants from State and national governmental organizations and from the Volkswagen foundation, the Eberhardt foundation, from Eli Lilly Foundation, from Janssen and from Celltech/USB. He was involved in clinical trials with Janssen, Medice, Lilly, Astra Zeneca and serves on a DSMB for Pfizer. He got travel grants from or served as a consultant for Aventis, Bayer, Bristol-MS, J&J, Celltech/USB, Lilly, Medice, Novartis, Pfizer, Ratiopharm, Sanofi-Synthelabo; VFA & Generikaverband, the Vatican, NIMH, AACAP, DFG, EU and European Academy. No shares and no direct affiliation with a pharmaceutical company.

## Authors' contributions

All authors have contributed essential parts to the manuscript and are entirely responsible for the scientific content of it. MK and JMF planed the study. MK and US conducted the interviews. HS, JB, AP produced the transcriptions and/or conducted the analysis. MK created and edited the drafts. JMF edited the last draft. All authors approved the final manuscript.
